# Sex-specific performance, trade-offs and trait repeatability across the lifetime of the world's largest semelparous mammal (*Dasyurus hallucatus*)

**DOI:** 10.1242/jeb.249969

**Published:** 2025-04-07

**Authors:** Gabriella R. Sparkes, Jaime Heiniger, Nicholas M. A. Smith, Vincent Careau, Ami F. Amir Abdul Nasir, Skye F. Cameron, Robbie S. Wilson

**Affiliations:** ^1^ School of the Environment, University of Queensland, St Lucia, QLD 4072, Australia; ^2^ School of Environmental and Rural Science, University of New England, Armidale, NSW 2351, Australia; ^3^ Institut de Systématique, Evolution, Biodiversité (ISYEB) EPHE-PSL, Université PSL, MNHN, CNRS, SU, UA 57 rue Cuvier, 75005 Paris, France; ^4^ Department of Biology, University of Ottawa, 30 Marie Curie, Ottawa, ON, Canada, K1N 6N5; ^5^ Australian Wildlife Conservancy, Subiaco East, WA 6008, Australia

**Keywords:** Semelparity, Sexual dimorphism, Sprint speed, Bite force, Life history

## Abstract

The activities that define survival and reproductive success in animals depend on their physical performance. However, performance is a complex trait, and organisms must balance competing demands of multiple underlying factors every time they undertake an activity. For example, the morphology that increases bite force (i.e. increased head size) – improving fighting ability – should constrain sprinting performance by adding mass to the body. Consequently, trade-offs between fighting and escape performance might be sex specific where sexual dimorphism is present, or pronounced in animals with extreme breeding strategies. Northern quolls (*Dasyurus hallucatus*) are a sexually dimorphic marsupial, with sex-specific life history strategies; males die after a single synchronous breeding season, while females can live and breed for 2–3 years. We investigated the effects of sex and life history on whole-animal performance and assessed whether sprint speed and bite force trade off among or within individual male and female quolls. We used a repeated measures dataset spanning 3 years. We identified significant sex differences in morphology and performance, notably after breeding, where male sprint speed decreases but female bite force increases. Both body size and body condition were strong predictors of performance. However, we found no trade-off between sprint speed and bite force, suggesting that ecologically relevant tasks for survival and reproduction – fighting capacity and escape ability – may evolve independently in both male and female northern quolls. Finally, we assessed the repeatability of morphological and performance traits and demonstrated the importance of study design when quantifying variance in animal performance, especially for animals with complex life histories.

## Teaser

**Summary:** A 3 year dataset investigating northern quoll performance reveals that extreme reproductive strategies drive sex-specific changes in sprint speed and bite force, with no trade-off, suggesting these traits evolve independently in both sexes.

## INTRODUCTION

Movement underlies all animal behaviour. Animals must move to find mates, avoid predators, forage, travel between patches and defend territories. Success in any of these activities has consequences for an individual's fitness – their ability to grow, survive and reproduce (Holyoak et al., 2008). But it is ultimately an individual's performance in whole-animal functions such as speed, strength, endurance, agility and motor control that determines their success in these activities (Husak and Lailvaux, 2014; Irschick et al., 2008). Such traits are underpinned by a suite of morphological and physiological components, working simultaneously to facilitate complex movement. Yet, there are physical limits to success in any task. When multiple performance tasks rely on shared morphological features but impose conflicting demands, success in one trait comes at the expense of performance in another (Garland et al., 2022; Lailvaux and Husak, 2014). Animals must balance the competing demands of these factors, or ‘functional trade-offs’ every time they move (Angilletta et al., 2008; Clemente and Wilson, 2015; Higham et al., 2001; Irschick et al., 2005; Van Damme et al., 2002; Vanhooydonck et al., 2001; Wynn et al., 2015).

Functional trade-offs can manifest within individuals, among individuals of the same species, or between species (Dietz et al., 2007; James et al., 2005; Pasi and Carrier, 2003; Reidy et al., 2000; Vanhooydonck et al., 2014). Species vary in their ecological requirements and the functions they need to excel in to survive and reproduce, and these differences can be reflected in how trade-offs are expressed. For example, cheetahs (*Acinonyx jubatus*) are well adapted to rapid pursuit predation; their leg muscles contain a higher proportion of fast-twitch glycolytic muscle fibres than slow-twitch oxidative muscle fibres, which enables high acceleration and sprinting speeds but constrains locomotor endurance (Karlsson et al., 1981). At the morphological level, a cheetah's lean, elongated limbs and flexible spine also facilitate these high-speed, tortuous pursuits, allowing it to out-manoeuvre prey and conspecifics, but make it comparatively much weaker than a broad-chested, muscular African lion (*Panthera leo*) (Wilson et al., 2013).

Within a species, males and females often differ in the activities they need to perform in the wild to maximise survival and reproductive success. Each sex, therefore, experiences different selective regimes, which can drive divergence in the optimal phenotype required to maximally perform such activities (Murren, 2012; Simon et al., 2022). For example, in males of the common house gecko (*Hemidactylus frenatus*), selection on the whole-animal traits that underpin fighting behaviour (i.e. bite force) drives selection for the underlying functional trait (i.e. head size) (Huyghe et al., 2005). As a result, males with bigger heads have a greater fighting capacity, increasing mate acquisition and reproductive success (Lailvaux et al., 2019). However, the fighting advantage accrued by larger heads can reduce the speed at which an animal can sprint, climb and turn, as a result of the added weight of the head imposed on the body (Cameron et al., 2013; Herrel and Bonneaud, 2012; Wynn et al., 2015). While female *H. frenatus* also have disproportionately large heads as a result of sexual selection, they have developed longer hindlimbs to compensate for the cost of a larger head. Through alternative resource allocation, females circumvent the trade-off, avoid the locomotor cost and ultimately diverge from males in optimal phenotype (Cameron et al., 2013).

The consequences of sex-specific resource allocation in terms of whole-animal performance and functional trade-offs may be even more pronounced in animals that exhibit extreme breeding strategies (Orr and Garland, 2017). Semelparity is an extreme life-history strategy characterised by a single, highly synchronised breeding event with complete post-copulation mortality (Braithwaite and Lee, 1979). For some species, semelparity is exhibited in only one sex while the other reproduces multiple times, leading to divergence in optimal phenotype for performance. Semelparity has evolved in several invertebrate species (Hughes, 2017), but is rare among vertebrates, evolving only once in mammals – in members of the *Dasyurid* family (Hayes et al., 2019; Oakwood et al., 2001). Selection for ‘suicidal reproduction’ in these populations favours males that maximise their reproductive success by investing heavily in breeding traits, but to the detriment of their survival beyond the first breeding event. In contrast, females of these species may be iteroparous – allocating resources towards survival and maintenance to ensure their longevity for multiple breeding events (Fisher and Cockburn, 2006; Gaschk et al., 2023). Extreme differences in breeding strategies between males and females may reveal the importance of different traits in facilitating survival or reproductive success. Yet, how sex-specific breeding strategies affect whole-animal performance, the expression of functional trade-offs and the repeatability of performance across growth and reproduction has not been explored in a population of semelparous wild animals.

Northern quolls (*Dasyurus hallucatus*) provide a unique system to explore how differences between males and females influence performance and the expression of functional trade-offs. Male northern quolls are the largest semelparous mammal in the world – though more accurately, they are considered ‘facultatively semelparous’ as most, but not all, males die before 1 year of age (Hernandez-Santin et al., 2016; Oakwood et al., 2001). Before the first breeding event, males rapidly increase in body size and mass, and during the breeding season they fight extensively with other males and increase their home ranges 5-fold to increase mating opportunities (Heiniger et al., 2020). However, meeting these metabolic demands comes at a cost: males have less sleep (Gaschk et al., 2023) and suffer extreme physiological deterioration, a dramatic decline in immunity and rapidly lose weight, fur and condition until death (Oakwood et al., 2001). In contrast, female northern quolls can live up to 3 years and exhibit a highly promiscuous breeding strategy, mating with multiple males each breeding season. During this time, females maintain a constant home range (Heiniger et al., 2020) but become more aggressive in defence and competition with other females for resources (Oakwood, 2002). While technically iteroparous, females also experience this high cost of reproduction, typically with less than half of the population surviving to the next breeding season (Heiniger et al., 2020; Oakwood, 2002; Braithwait and Griffiths, 1994). These sex differences in life-history traits should theoretically drive differences in performance during tasks that facilitate survival and successful reproduction such as escape, chase and fighting capacities.

Investigating performance trade-offs in wild animals is a complicated task: traits must be ecologically relevant, repeatable over time, measurable at the whole-animal level and performed with maximal motivation (Irschick, 2003). Past research has relied on maximum performance values at limited time points to quantify functional trade-offs, often yielding spurious results (Herrel and Bonneaud, 2012). This is because, when phenotypic correlations are analysed as one source of variance rather than separately at the among- and within-individual levels, the expression of trade-offs may be masked (Careau and Wilson, 2017a; Simon et al., 2022). The detection of trade-offs may be further complicated by the multivariate and complex long-term effects of sex, ageing and breeding strategies on performance (Lailvaux et al., 2019). Short-term studies often fail to capture temporal variation and changes in performance over an individual's lifetime, because the repeatability of performance traits can be influenced by changes in intrinsic (e.g. hormonal state, motivation, diet, training, fat reserves) and extrinsic conditions (e.g. temperature, rainfall, food availability) (Araya-Ajoy et al., 2015; Careau and Wilson, 2017b). Therefore, longitudinal experimental design and variance partitioning approaches are essential for assessing trade-offs among performance traits. Multivariate mixed models (MMMs) can be used to simultaneously analyse repeated measures of performance, assess the repeatability of multiple performance traits across multiple time points, and partition trait correlations at the within- and among-individual levels (Araya-Ajoy et al., 2015; Berberi and Careau, 2019; Careau and Wilson, 2017a; Dingemanse and Dochtermann, 2013; Lailvaux et al., 2019).

In this study, we investigated the effects of sex differences in life-history and breeding strategies on performance in whole-organism traits – sprint speed and bite force – in a wild population of northern quolls. This study was conducted over 3 years (2012–2014), spanning three distinct phases of the quoll breeding season each year: pre-breeding, breeding and post-breeding. We took repeated measures of performance and morphological traits to assess the effects of limb length, head size, mass, overall body size and shape on sprinting and biting performance of males and females separately. We used MMMs to assess performance trade-offs between sprint speed and bite force by partitioning the (co)variance at the among- and within-individual levels. Finally, to explore the temporal variance of performance traits, we used our high recapture rates to estimate the repeatability of male and female performance traits across days, seasons, years and lifetimes. We hypothesised that sexual dimorphism observed in northern quolls is driven by sexual selection, and predicted that males produce stronger bite forces and faster sprint speeds than females. We also hypothesised that the male semelparous life-history strategy imposes intense physiological demands and predicted that male – but not female – performance declines after the breeding season as their body condition deteriorates. Finally, we hypothesised that a trade-off between sprinting and biting performance poses conflicting demands on the required morphology and physiology, and predicted negative correlations at both among- and within-individual levels in both sexes.

## MATERIALS AND METHODS

### Study site

Northern quolls (*Dasyurus hallucatus* Gould 1842) were captured on Groote Eylandt in the Northern Territory, Australia (13°50′8.91′′S, 136°25′3.10′′E) over a 3 year period from 2012 to 2014 (Heiniger et al., 2020). We used mark–recapture strategies and baited cage traps (20×20×60 cm; Tomahawk ID-103, Hazelhurst, WI, USA) to catch male and female quolls within a 128 ha permanent study site, across five trapping grids (540×300 m). Each grid contained 40 cage traps placed at 60 m intervals along four transect lines, 100 m apart. We microchipped (Trovan nano-transponder ID-100, Keysborough, VIC, Australia) and ear-tagged (1005-1 MONEL, National Band and Tag Co.) each quoll for identification in subsequent captures. Quolls were captured during time periods associated with important life stages each year of the study: the pre-breeding period (May–June), breeding period (July–August) and post-breeding period (September–October) (Heiniger et al., 2020). All research methodologies were approved by the University of Queensland's Animal Ethics Committee (SBS/014/11/AC and SBS/404/12/APA) and the Northern Territory Parks and Wildlife Commission (Permit number 44346). This research was conducted with permission from the Traditional Owners of Groote Eylandt, specifically the Bara Clan, who allowed access to their land. An Anindilyakwa Indigenous Protected Area Research Approval permit from the Anindilyakwa Land Council of Groote Eylandt was obtained for this research.

**Fig. 1.**
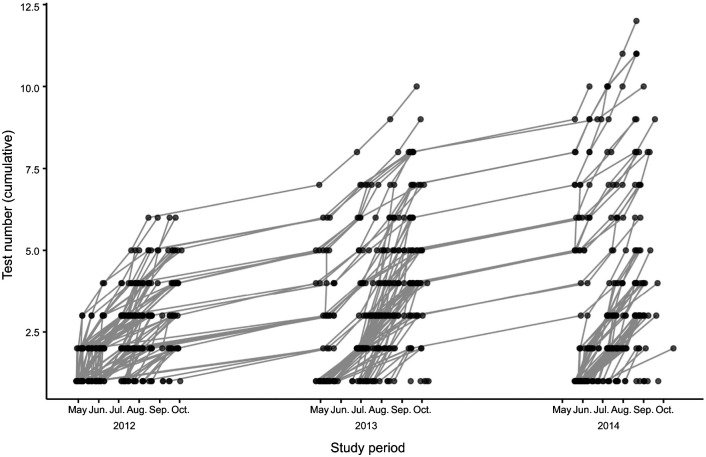
**Temporal distribution of repeated performance tests conducted on wild northern quolls (*Dasyurus hallucatus*) on Groote Eylandt, Northern Territory, Australia, between 2012 and 2014.** Successive tests made on a given individual (circles) are connected by a grey line, showing the cumulative test number for different individuals that were repeatedly sampled throughout the testing period (May–October) each year (*n*=322 individuals: 165 females, 157 males).

During each capture, morphological and performance measurements were taken for each quoll at the Anindilyakwa Land and Sea Rangers' facilities. Quolls were released before sunset on the same day at the site of capture. A total of 322 quolls (165 females, 157 males) were captured and tested at least once for at least one set of morphological or performance (sprint speed and bite force) measurements. Because of the expanded habitat ranges of males during the breeding season and their mortality rate after breeding, some males were captured and tested only once. In contrast, some females were captured repeatedly throughout the entire breeding season (May–October) and/or across the entire duration of the study (2012–2014; Fig. 1). On average, we tested each quoll 2.8 times, with the total number of tests per individual ranging from 1 to 12.

### Morphology

All measurements were taken within 6 h of capture. We estimated age based on molar condition and reproductive stage (Heiniger et al., 2020). We measured body mass using digital scales (±0.1 g; A&D Company Limited HL200i, Brisbane, QLD, Australia). Morphological measurements were taken using either digital callipers or tailor's tape (±1 mm; Whitworth, Brisbane, QLD, Australia) and included head length, head width, body length, tail length, tail width, hindfoot length, hindlimb length and forelimb length (see Wynn et al., 2015, for full measurement details). Morphological measurements were taken 3 times per trait and averaged to reduce measurement error. Principal component analysis (PCA) was conducted on all eight morphological traits to obtain a single measure of overall body size. The first component, PC_bodysize_, explained 73.67% of the variation in morphology, with all vectors loading in a positive direction, suggesting a measure of overall body size (Table 1). There was a strong positive correlation between body size and mass (*R*^2^=0.85). We used PC_bodysize_ in analyses of both sprint speed and bite force because it captures a more whole-animal view of the effects of size on performance. The second component, PC_condition_, explained 7.9% of the variation in morphology. Tail diameter accounted for most of the variation in this second component. Because quolls are known to store fat in the base of their tails, tail diameter has previously been used as a reference for body condition (Serena and Soderquist, 1988). Therefore, we used PC_condition_ as a measure of body condition in the models (Table 1). For both sexes, body size and condition can be highly variable through maturity and during different stages of the breeding season (Oakwood et al., 2001). For this reason, taking morphological measurements at each capture was necessary to record changes across the study period, as these changes could influence individual performance.

**Table 1.** Principal component analysis matrix and means and standard deviations of the eight morphological variables measured for male and female northern quolls (*Dasyurus hallucatus*)

Morphological trait	Measurement (mm)	PC_bodysize_	PC_condition_
Percentage variance		73.67	7.90
Body length	175.76±18.96	0.35	0.22
Tail length	220.82±23.44	0.30	−0.56
Tail diameter	13.94±2.19	0.30	0.70
Foot length (left)	38.76±2.65	0.35	−0.32
Hindlimb length (left)	61.62±4.34	0.39	−0.09
Forelimb length (left)	48.77±4.06	0.39	−0.09
Head width	36.12±2.89	0.37	0.19
Head length	67.23±4.21	0.36	−0.02

The means and standard deviations of each measured variable, factor loadings, direction in which they contribute towards the principal components (PC) and the percentage of variance explained by the two main PCs. Measurements were taken as per Wynn et al. (2015).

### Sprint speed

We assessed sprint speed using motion-capture cameras (Optitrack S250e, NaturalPoint Inc., Corvallis, OR, USA) positioned alongside and 2 m above a linear racetrack (4×0.6 m, rubber substrate for traction). Cameras were connected to ‘Motive’ motion-capture software, filming at 125 Hz. Quolls were chased back and forth along the full extent of the racetrack a minimum of 3 times, to capture 6 measures of maximum sprint speed. We recorded maximum sprint speed from the central 2 m of the track to allow quolls to reach maximum speed during each lap. Videos were analysed frame by frame using TRACKER software (Open Source Physics, Boston, MA, USA) to quantify instantaneous maximum sprint speed for each trial number (see Supplementary Materials and Methods for more details).

### Bite force

We quantified bite force using a custom-built force transducer (see Supplementary Materials and Methods for more details). We calibrated the force transducer daily with weights of known mass up to a force greater than any quoll could produce in a maximum bite. When restrained, quolls readily bit down onto anything near their mouths, so individuals were easily encouraged to bite down on the metal plates of the force transducer for approximately 15 s. Three separate bites were recorded per quoll. We extracted the force (N) measurements using PowerLab software (ADInstruments, Sydney, NSW, Australia). Quolls were given adequate rest between testing activities to ensure sufficient muscle recovery and to maintain maximal motivation.

### Statistical analyses

#### Univariate models considering repeated measures

All analyses were performed in R version 4.3.0 (http://www.R-project.org/). All univariate and bivariate models were run using ASReml-R version 4 (https://vsni.co.uk/software/asreml-r/) and the significance of fixed effects on performance traits was tested using Wald tests. Sex-specific univariate models were first run separately for each performance trait – sprint speed and bite force – including all repeated tests made on a given individual. Body size (PC_bodysize_ as it explained most of the variance in morphology), body condition (PC_condition_), age (excluded from male models as none survived past 1 year of age) and reproductive season (pre-breeding, breeding or post-breeding) were fitted as fixed effects. Age and reproductive season were fitted as factors with three levels in each. Test day (the number and order of captures throughout the entire study) and trial number [the order of sprint trials (i.e. 1–6) or bite (i.e. 1–3) trials conducted per test day] were also fitted as fixed effects to account for their influence on sprint speed and bite force separately. Sprint speed, bite force and measures of body mass and size were *z*-transformed (mean=0, variance=1) to assist in model convergence and to enable standardised variables to be interpreted accurately. To explore changes in body condition, we also ran a univariate model considering the effects of body size, age, sex and reproductive season on body condition in males and females.

#### Performance across reproductive season

We ran a separate linear mixed-effects model (*n=*34 females, 48 males) including only individuals measured at least once consecutively in each reproductive season (pre-breeding, breeding, post-breeding) to assess the effects of reproductive status on bite force and sprint speed separately. Of this group, we included first-year male and female quolls to control for age as a confounding variable. These models were fitted using the lme4 package and general linear hypothesis tests were performed using the multcomp package in R to determine whether sprint speed and bite force significantly differed between reproductive seasons (Bates et al., 2015).

#### Repeatability

Repeatability measures estimate the proportion of total phenotypic variance attributable to among-individual (*V*_ind_) versus within-individual (*V*_e_) differences (Araya-Ajoy et al., 2015). Because individuals were repeatedly measured across multiple time points, repeatability can be estimated across multiple temporal scales. We included individual identity (ID) as a random effect in all univariate models to test among-individual variance – that is, whether individual quolls consistently differed in performance for each trait. We used this variable to estimate an individual's repeatability across all measures during the entire study period; this is a measure of long-term repeatability (Eqns 1 and 5; *R*_long-term_). Expanding on this, we used a unique combination of ID and the year of the study (ID_YEAR) to group all observations made on an individual each year and estimate an individual's performance trait repeatability across years (Eqn 2; *R*_year_). We included this measure only for females because all males died within 1 year. We also combined ID with reproductive season (pre-breeding, breeding, post-breeding) to estimate an individual's performance trait repeatability across seasons (Eqns 3 and 6; *R*_season_). For females, we nested season within age because a female could potentially be measured in the pre-breeding, breeding and/or post-breeding season in the first, second and/or third year (females, ID_YEAR_SEASON; males, ID_SEASON). This combination groups all observations made on an individual within a given season (for males) and within a season each year (for females). Finally, we combined ID and Julian Day. Julian Day represents the number of days since 1 January 2012, and corresponds to each test day throughout the duration of the study. We nested ID and Julian Day within year for females (ID_YEAR_DAY) and within season for males (ID_SEASON_DAY), so that all repeated measures taken for sprint speed and bite force were grouped for a given individual on a given day (test). This represents a measure of short-term repeatability across days (Eqns 4 and 7; *R*_short-term_). Together, these terms capture the temporal dependency of observations and enable a detailed partitioning of repeatability over the entire study period, 2012–2014. We expected performance repeatability measures to decrease from short-term to long-term, as repeated measures taken on a given individual, temporally close to one another, are likely to experience similar environmental conditions impacting the phenotype, resulting in higher repeatability estimates. We also estimated the repeatability of body size, mass and body condition at long-term (study duration), yearly (females only) and seasonal scales, for comparison with the performance repeatability estimates at those same scales (Table S3). Repeatability estimates and the upper and lower 95% confidence intervals (CIs) were calculated using the varcomp function in ASReml-R (https://vsni.co.uk/software/asreml-r/).

Equations for repeatability estimates for females were as follows:
(1)

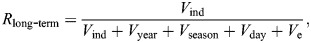

(2)

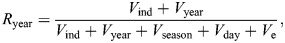

(3)

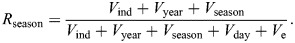

(4)

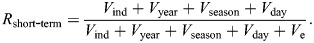


Equations for repeatability estimates for males were as follows:
(5)

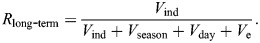

(6)

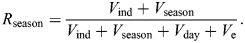

(7)

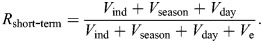


#### Bivariate models for trade-off detection

To test for the presence of a trade-off between sprint speed and bite force, we ran sex-specific bivariate mixed-effects models. We estimated the phenotypic correlation between sprint speed and bite force by partitioning the phenotypic (co)variance at the among-individual and within-individual levels (Dingemanse and Dochtermann, 2013). A negative correlation indicates that an increase in the performance of one trait directly constrains the performance of another, suggesting a trade-off. For this model, the maximum values of sprint speed and bite force from all repeated measures for each individual on a given day were *z*-transformed (mean=0, standard deviation=1). This approach differs from the ‘personal best’ method, which uses a single maximum value to represent an individual's maximum performance across all tests and conditions (Careau and Wilson, 2017b). Instead, this approach considers all tests made on a given individual on a given day, retaining the trial that yielded the highest value within that day. Age (females only), reproductive status, body size (PC_bodysize_) and body condition (PC_condition_) were included as fixed effects in this model, with ID as a random effect. The bivariate models were fitted using a correlation matrix at the among-individual and residual levels, which provided estimates for the among-individual correlation (*r*_ind_) and within-individual correlation (*r*_e_). We used profile likelihoods using the nadiv package (Wolak, 2012) to calculate the upper and lower 95% CIs. The best linear unbiased predictors (BLUPs) and standard errors (s.e.) for each individual were extracted from the bivariate models and plotted for visualisation (see Results, ‘Bivariate models for trade-off detection’). BLUPs are useful in visualising random effects estimates and correlations among traits (Lailvaux et al., 2019; Newar and Careau, 2018).

## RESULTS

### Sprint speed

**Fig. 2.**
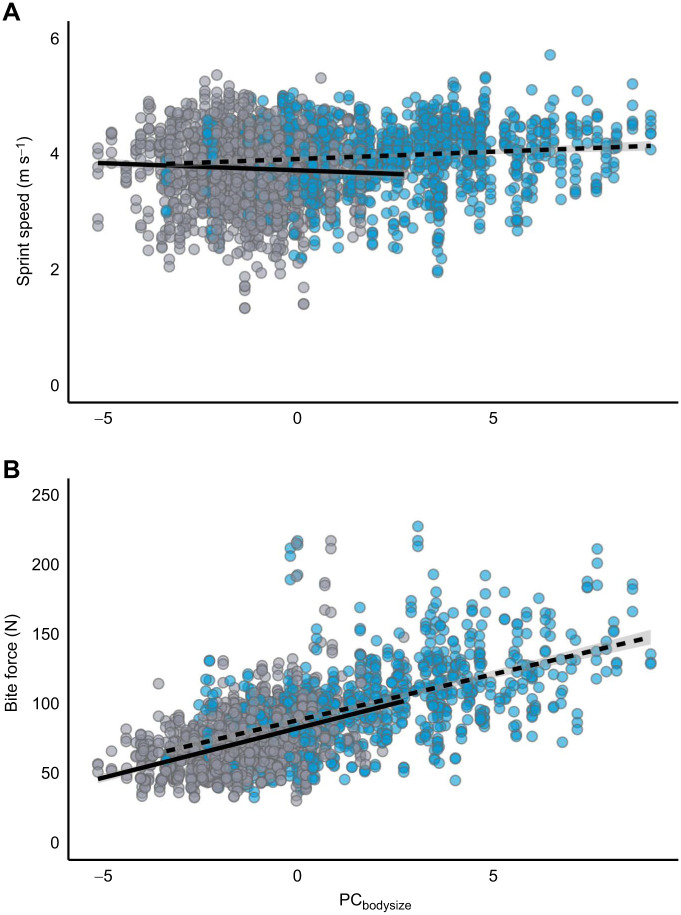
**Sprint speed and bite force in wild female and male northern quolls (*D. hallucatus*).** Sprint speed (A) and bite force (B) are shown as a function of PC_bodysize_ (grey circles, females, *n*=165; blue circles, males, *n*=157). Partial residuals for six measures of sprint speed and three measures of bite force for each individual adjusted for the other fixed and random effects contained in the univariate linear mixed effects models. Regression lines show the relationship between body mass and performance traits for each sex (females, solid lines; males, dashed lines), with *P*-values represented in Table 2.

We analysed sprint speed for 321 quolls (164 females, 157 males) using univariate mixed models that included all repeated trials (i.e. repeated measures within a test day) across all test days (*n*=1926 trials). Overall, larger and heavier males sprinted faster (Table 2, Fig. 2A; *F*_male_*=*5.76, d.f._male_*=*1138.2; *P*_male_=0.02). However, sprint speed was not affected by body size for females (Table 2, Fig. 2A; *F*_female_=0.1, d.f._female_*=*1230; *P*_female_*=*0.75). Sprint speed was not affected by body condition for males or females (Table 2; *F*_male_*=*0.82, d.f._male_*=*1, 217.1; *P*_male_*=*0.37; *F*_female_*=*0.03, d.f._female_*=*1288.8; *P*_female_*=*0.86). Age did not affect sprint speed for females (Table 2; *F*_female_*=*1.59, d.f._female_*=*1223.1*; P*_female_*=*0.21). Within each test day, sprint speed significantly increased with successive trials, on average by 0.27 m s^−1^ for females and 0.11 m s^−1^ for males from trial 1 to 6 (Table 2; *F*_female_=164.4, d.f._female_=11,505; *P*_female_*<*0.001; *F*_male_=26.46, d.f._male_=11,112.2; *P*_male_*<*0.001). Between testing days, sprint speed did not differ significantly for males or females (Table 2; *F*_male_*=*2.79, d.f._male_*=*1, 217.4; *P*_male_*=*0.10; *F*_female_*=*2.60, d.f._female_*=*1250.1; *P*_female_*=*0.11). We also tested for sexual dimorphism in the population and found that males were 43% larger and 31% heavier than females (Table S4; males=486±144 g, females=337±71 g).

**Table 2.** Summary statistics (estimates and 95% confidence intervals, CI) from two separate univariate models including all trials of sprint speed and bite force (both standardised to a mean of 0 and a variance of 1) in wild female and male northern quolls (*D. hallucatus*)

	Sprint speed			Bite force		
	Estimate	95% CI		Estimate	95% CI	
		Lower	Upper		Lower	Upper
Females				** **	** **	** **
Fixed effects				** **	** **	** **
PC_bodysize_	−0.02	−0.11	0.08	**0**.19***	**0**.**13**	**0**.**25**
PC_condition_	−0.01	−0.16	0.13	**0**.22***	**0**.**13**	**0**.**32**
Age	0.19	−0.11	0.48	0.05	−0.14	0.23
Season						
Breeding	−0.14	−0.33	0.05	**−0**.24***	**−0**.**37**	**−0**.**11**
Post-breeding	0.03	−0.25	0.32	**0**.**30**	**0**.**09**	**0**.**51**
Test no.	−0.05	−0.12	0.01	0.01	−0.03	0.05
Trial no.	**0**.11***	**0**.**10**	**0**.**13**	**−0**.16***	**−0**.**17**	**−0**.**15**
Males	** **	** **	** **	** **	** **	** **
Fixed effects	** **	** **	** **	** **	** **	** **
PC_bodysize_	**0**.06*	**0**.**01**	**0**.**11**	**0**.23***	**0**.**19**	**0**.**27**
PC_condition_	0.06	−0.07	0.19	0.06	−0.06	0.18
Season						
Breeding	0.02	−0.19	0.23	−0.08	−0.32	0.15
Post-breeding	−0.10	−0.50	0.31	0.20	−0.15	0.55
Test no.	−0.11	−0.23	0.01	−0.10	−0.21	0.00
Trial no.	**0**.05***	**0**.**03**	**0**.**07**	**−0**.22***	**−0**.**24**	**−0**.**21**

Fixed effects included in the univariate models include body size (PC_bodysize_), body condition (PC_condition_), age [1, 2 or 3 years old (females only)], season [pre-breeding, breeding or post-breeding], test number and trial number. Significant fixed effect estimates are in bold; *P<0.01; ***P=0.00.

### Bite force

We analysed bite force for 321 quolls (164 females, 157 males) using a univariate mixed model that included all repeated measures made on a given individual (*n=*963 trials). Male quolls produced greater bite forces than females, with males exerting 28% greater force (Table S4; *t=*−13.68, d.f.=1482.13; *P<*0.001). Bite force increased with body size for both females and males (Table 2, Fig. 2B; *F*_female_=41.05, d.f._female_*=*1225.5; *P*_female_*<*0.001; *F*_male_=131.8, d.f._male_*=*1,96.7; *P*_male_*<*0.001). Body condition did not affect bite force for males (*F*_male_=1.01, d.f._male_*=*1193; *P*_male_*=*0.30), but females with higher body condition had significantly greater bite force (*F*_female_=21.46, d.f._female_*=*1312.1; *P*_female_*<*0.001). Bite force was not affected by age for females (Table 2; *F*_female_*=*0.24, d.f._female_*=*1242.8; *P*_female_*=*0.62). Bite force significantly decreased with each successive trial within a test day, on average by 9.3 N for females and 13.1 N for males between trials 1 and 3 (Table 2; *F*_female_=676.9, d.f._female_*=*1818.2; *P*_female_*<*0.001; *F*_male_=676.5, d.f._male_*=*1565.3; *P*_male_*<*0.001). However, bite force did not differ between test days for either females or males (Table 2; *F*_female_=0.09, d.f._female*=*_1267.5; *P*_female_*=*0.77; *F*_male_*=*3.63, d.f._male_=1174.1; *P*_male_=0.06).

### Body condition

Northern quolls mature rapidly before breeding and males experience senescence quickly after breeding. Because body condition fluctuates dramatically over the lifetime of a quoll and reflects the general health and energy reserves available, we investigated how changes in body condition were affected by body size (PC_bodysize_), sex, reproductive season, test number and the interaction between sex and reproductive season in a univariate model. We found that all variables excluding test number had a significant effect on the body condition of quolls. Body condition increased with body size (Table S5; *F*=9.43, d.f.=1628.5, *P*<0.001). Males, on average, had worse body condition than females (Table S5; *F*=71.03, d.f.*=*1377.2, *P*<0.001). Body condition correlated negatively with reproductive season for both sexes (Table S5; *F=*14.46, d.f.=1853.4, *P<*0.001). Males experienced a greater decline in body condition over reproductive season than females (Table S5; *F=*56.17, d.f.=1846.4, *P*<0.001).

### Performance across reproductive season

**Fig. 3.**
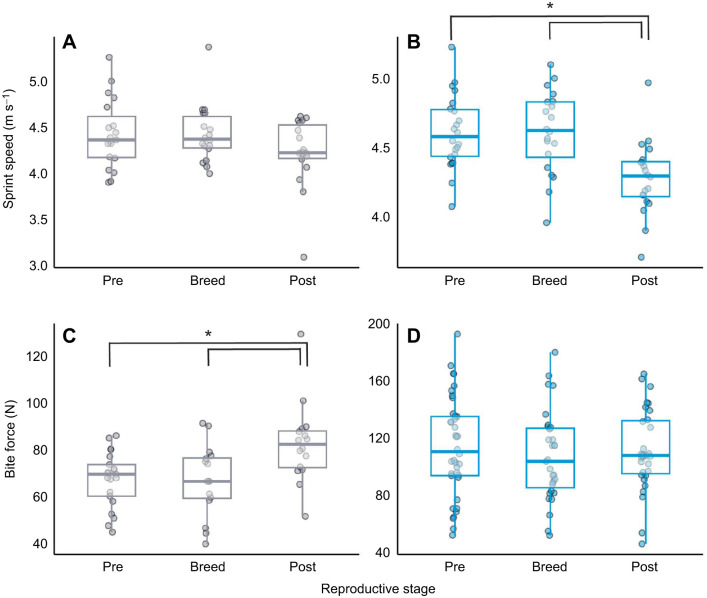
**Sprint speed and bite force across the breeding season in wild female and male northern quolls (*D. hallucatus*).** Sprint speed (A,B) and bite force (C,D) are shown for pre-breeding, breeding and post-breeding stages for first-year females (left; grey; *n*=34) and males (right; blue; *n=*48) that were measured consecutively across each season. Box plots show medians, upper and lower quartiles and 1.5× interquartile range. Significantly different performances from the linear mixed-effects model are marked by brackets and asterisks; *P<0.01. Unmarked groups are not statistically different.

The effects of reproductive season on performance measures varied by sex in the univariate models considering all trials across all years. For males, sprint speed and bite force did not significantly change across season (Table 2; sprint speed: *F*_male_=0.41, d.f._male_*=*2169.5; *P*_male_=0.67; bite force: *F*_male_=1.93, d.f._male_*=*2254.1; *P*_male_=0.15). For females, reproductive season significantly affected bite force (Table 2; bite force: *F*_female_=15.65, d.f._female_*=*2387.3; *P*_female_<0.001) but not sprint speed (Table 2; sprint speed: *F*_female_=1.64, d.f._female_*=*2254.6; *P*_female_=0.20). We then ran separate tests for sprint speed (*n*=18 females, 20 males) and bite force (*n*=16 females, 28 males) focusing only on first-year quolls captured in every season consecutively (pre-breeding, breeding, post-breeding), to remove age as a confounding variable. The model was run using maximum values from repeated measures per testing session. There was no significant effect of season on bite force for males (Table S1; Fig. 3D; *F*_male_=1.02, d.f._male_=2,70.84; *P*_male_=0.37), or on sprint speed for females (Table S1; Fig. 3A; *F*_female_=0.77, d.f._female_=2,40.83; *P*_female_=0.47). However, we found that male quolls sprinted significantly slower in the post-breeding season compared with the pre-breeding and breeding season (Table S1; Fig. 3B; *F*_male_*=*5.00; d.f._male_=2,45.59; *P*_male_=0.01). We also found that females produced significantly stronger bite forces in the post-breeding season (Table S1; Fig. 3C; *F*_female_=4.49, d.f._female_=2,37.21; *P*_female_=0.02).

### Repeatability

While accounting for the fixed effects above, we calculated repeatability estimates across individuals over the duration of the entire study (*R*_long-term_), across years (*R*_year_, females only), across seasons (*R*_season_; pre-breeding, breeding, post-breeding) and across testing days (*R*_short-term_). Sprint speed was significantly repeatable across all temporal scales for both sexes (Table 3). Long-term repeatability estimates show that 28% and 39% of the phenotypic variance in sprint speed performance for females and males, respectively, was attributable to among-individual variance. Year and season did not explain significant amounts of the variability in performance, and as a result repeatability within years and reproductive seasons for females did not differ from that of the long-term estimate (*R*_long-term_=*R*_year_=*R*_season_=0.28; Table 3). Sprint speed was more repeatable within reproductive season for males (*R*_season_=0.54). Finally, sprint speed was most repeatable on a short-term scale for both females and males (females *R*_short-term_=0.58; males *R*_short-term_=0.62).

**Table 3.** Repeatability estimates (*R*) and 95% CIs for sprint speed and bite force at each temporal scale extracted from univariate models including all trials of each performance measure for wild female and male northern quolls (*D. hallucatus*)

	Sprint speed			Bite force		
	Estimate	95% CI		Estimate	95% CI	
		Lower	Upper		Lower	Upper
Females						
Variance components						
*V*_ind_	0.28	0.15	0.40	0.07	0.01	0.13
*V*_year_	<0.01	<0.01	<0.01	0.02	−0.04	0.09
*V*_season_	<0.01	<0.01	<0.01	0.01	−0.06	0.08
*V*_day_	0.29	0.21	0.37	0.25	0.18	0.32
*V*_e_	0.42	0.39	0.45	0.03	0.03	0.03
Repeatability						
*R*_long-term_	0.28	0.18	0.38	0.18	0.03	0.34
*R*_year_	0.28	0.18	0.38	0.24	0.12	0.37
*R*_season_	0.28	0.18	0.38	0.26	0.09	0.44
*R*_short-term_	0.58	0.52	0.63	0.92	0.91	0.93
Males						
Variance components						
*V*_ind_	0.34	0.20	0.48	0.02	−0.11	0.12
*V*_season_	0.13	0.01	0.26	0.03	−0.21	0.27
*V*_day_	0.07	−0.03	0.17	0.71	0.47	0.95
*V*_e_	0.34	0.31	0.37	0.04	0.04	0.05
Repeatability						
*R*_long-term_	0.39	0.27	0.50	0.02	−0.14	0.15
*R*_season_	0.54	0.40	0.67	0.04	−0.23	0.32
*R*_short-term_	0.62	0.56	0.68	0.95	0.94	0.96

Repeatability of performance traits at each temporal scale was calculated using variance components extracted from each univariate model. Variance components of the random effects included in the models: individual identity (*V*_ind_), age (*V*_year_ – females only), season (*V*_season_), day of test number (*V*_day_) and residual (*V*_e_).

Repeatability estimates for bite force also increased from long term to short term, as expected (Table 3). Long-term repeatability estimates show that only 18% and 2% of the phenotypic variance in bite force performance for females and males, respectively, was attributable to among-individual variance. Bite force repeatability within season for males was also low (*R*_season_=0.04). Bite force was moderately repeatable for females at the long-term, yearly and seasonal scales (females: *R*_long-term_=0.18, *R*_year_=0.24, *R*_season_=0.26), and highly repeatable on a short-term scale for both females (*R*_short-term_=0.92) and males (*R*_short-term_=0.95).

We also estimated the repeatability of body size (PC_bodysize_) across the duration of the study, across years (females only) and across seasons (Table S3). We found similar trends, as expected, where measures of body size were highly repeatable at shorter time scales (Table S3; females *R*_season_=0.91, *R*_year_=0.84; males *R*_season_=0.99) and decreased as measures became temporally more distant (females *R*_long-term_=0.56; males *R*_long-term_=0.95). The high variance at *V*_ind_ indicates that there was a large amount of variance in body size among individuals, but little within, resulting in a high repeatability of body size across a male individual's lifetime.

### Bivariate models for trade-off detection

**Fig. 4.**
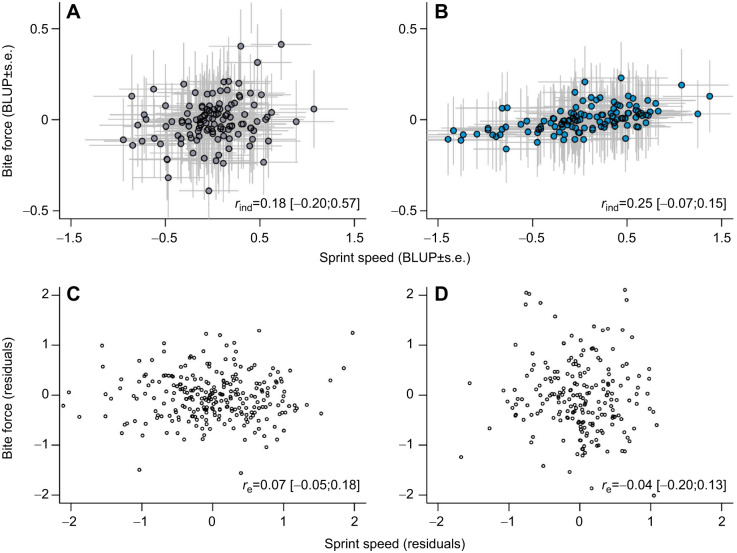
**Correlation between sprint speed and bite force in wild female and male northern quolls (*D. hallucatus*).** Among-individual correlations (*r*_ind_; A,B) and within-individual correlations (*r*_e_; C,D) between sprint speed and bite force are shown for females (left; *n=*122) and males (right; *n*=124) on Groote Eylandt, Northern Territory, Australia in 2012–2014. In A,B, best linear unbiased predictors (BLUPs; circles) extracted from a multivariate mixed model represent the individual deviations from the population mean, with standard errors (grey lines). C,D shows the residuals (open circles) for sprint speed and bite force. Groups are not statistically different.

We used sex-specific bivariate models that included all measures taken for each individual but retained the trial that yielded the highest value within each test day. This model allowed us to partition the variance and correlation between sprint speed and bite force at the among-individual and within-individual levels (*n*=122 females, 124 males). We found no evidence for a trade-off occurring between sprint speed and bite force for males or females either among individuals or within individuals (Fig. 4; females *r*_ind_=0.18 [−0.20;0.57], *r*_e_=0.07 [−0.05;0.18]; males *r*_ind_=0.25 [−0.07;0.15], *r*_e_=−0.04 [−0.20;0.13]). There was no correlation between sprint speed and bite force for either males or females, according to profile likelihood tests (Fig. S1). While sprint speed and bite force appeared to be positively correlated in Fig. 4A,B, the uncertainty, as determined by the confidence intervals, was too large to consider it significant. All main effects that were significant in the sex-specific univariate models remained significant in the sex-specific bivariate models as well (Table S2).

## DISCUSSION

In this study, we explored the complex interplay between morphology, performance, life history and sex in northern quolls (*D. hallucatus*), a species in which males and females vastly differ in breeding strategies. Male northern quolls were larger, heavier and produced stronger bite forces than females, but sprint speed did not differ between the sexes. Larger quolls produced greater bite forces for both sexes, and bigger males had slightly faster sprint speeds. Previous studies of northern quolls and other mammals also report that larger body sizes enhance strength-based performance tasks, such as bite force and grip strength, but not running performance such as sprint speed and acceleration (Berberi and Careau, 2019; Charters et al., 2018; Freeman and Lemen, 2008; Rew-Duffy et al., 2020; Tan and Wilson, 2011). Larger bodies enable proportionately larger muscle attachment sites and a greater force-producing capacity (Cameron et al., 2013; Huyghe et al., 2005; Lailvaux et al., 2019; Viacava et al., 2020). However, while increased body mass generally requires more effort to overcome inertia when accelerating and sprinting (Tan and Wilson, 2011), heavier animals may compensate for this limitation by ‘pre-stretching’ the limb muscles, allowing for more forceful contractions during acceleration from a standstill (Scales and Butler, 2007). This compensatory mechanism may explain why larger male quolls in our study had slightly higher sprint speeds despite their increased mass. Notably, overall body size alone accounted for most of the variance in sprint speed and bite force, rather than specific morphological traits such as head size or hindlimb length. Our findings support previous research suggesting that sexual dimorphism in this species occurs in overall body size rather than changes in the shape or proportion of specific morphological features (Charters et al., 2018; Viacava et al., 2020).

### Changes in performance across breeding season

The body condition and sprinting speed of male northern quolls markedly decreased across the breeding season, but interestingly, there was no change in body size or bite force. Male northern quolls use a ‘suicidal reproduction’ strategy in which there is only a single, short window of opportunity to mate with as many females as possible and then die shortly thereafter. In many semelparous species, the larger, more dominant males tend to sire more offspring and survive longer than smaller males (Fisher and Cockburn, 2006; Fisher et al., 2013). Therefore, to be competitive in this intense period, males seemingly invest as much as possible into being fast, fit, agile and strong, consequently diverting energy and time away from crucial physiological functions such as resting and immune responses (Gaschk et al., 2023; Hernandez-Santin et al., 2016). Our findings suggest that their intense activities across the breeding season and investment in their reproductive function results in a substantial reduction in their locomotor ability and body condition. However, larger male northern quolls maintain their size and strength throughout the breeding season, which may also allow them to outcompete conspecifics and secure more matings. Nevertheless, reproduction takes precedence over survival, and male northern quolls die on that hill. The specific mechanism driving their post-reproductive mortality is unclear (Oakwood et al., 2001), but the decline coincides with a peak in testosterone during breeding, and a post-breeding decline in immune markers, suggesting that immune suppression may contribute to their rapid physiological deterioration (Schmitt et al., 1989). In other semelparous dasyurids, elevated cortisol drives this mortality, but unlike these smaller marsupials, cortisol does not appear to be a primary driver of male quoll senescence (Schmitt et al., 1989). Instead, the post-breeding collapse of male northern quolls seems to be the consequence of a complex interplay between extreme energy expenditure, high testosterone and suppressed immune function.

Female northern quolls produced stronger bite forces in the post-breeding season and, in general, females with higher body condition produced stronger bite forces across all seasons. The increase in female bite force after breeding likely indicates an increase in aggression, a pattern often associated with breeding females, and may also be influenced by post-breeding hormonal changes. Heightened aggression and increased defensive behaviours, such as female–female fighting, have been identified in the females of many species at different stages of reproduction (Stockley and Campbell, 2013). Because they constantly encounter coercive males and competitive conspecifics, females with a strong fighting capacity – driven by a strong bite force – may be better equipped to defend territories, protect their young and fight for resources. Proficiency in these behaviours is likely to improve female individual fitness by maximising survival and reproductive success. Further, given that females can live and breed across 2–3 years and are the only parent to invest in the care of offspring, it seems females invest energy into being strong, healthy and aggressive to defend themselves and their young. Across many species, previous studies have found that females with higher body condition perform better in a range of whole-animal and reproductive performances (Orr and Garland, 2017), and in northern quolls, females with higher body condition reportedly have greater survival rates to 21 months (Rew-Duffy et al., 2020). These findings suggest high body condition, indicating high energy reserves, supports performance in whole-animal tasks such as bite force, maximising both longevity and fecundity in female northern quolls (Serena and Soderquist, 1988). Future research could profit from identifying the specific mechanisms driving mortality in this species, and how changes in hormones affect the whole-animal performance of both males and females.

### The (lack of a) trade-off between sprint speed and bite force

We found no evidence of a trade-off between sprint speed and bite force in male or female northern quolls, either among or within individuals. Although there was significant individual variance in these traits, there was no shared (co)variance, meaning that strong biters were not generally slow sprinters. We also found no evidence for compensatory mechanisms in either sex – meaning that longer hindlimbs do not appear to offset the locomotor cost of a large head for producing strong bite forces. Instead, our results suggest that selection for larger body sizes in general maximises bite force without constraining sprint speed. Further, each performance trait appears to confer a sex-specific advantage to survival or reproductive outcomes. Bite force and sprint speed, and their associated ecological tasks – fighting capacity and escaping or chasing ability – may evolve independently in this population of northern quolls.

The absence of a trade-off between sprinting and biting performance, however, does not preclude a trade-off between other functional traits. For example, previous studies have found trade-offs between bite force and motor control, and sprint speed and cornering speed (Charters et al., 2018; Wynn et al., 2015), suggesting that other functional groups may impose greater conflicting demands on northern quoll morphology than biting and sprinting in isolation. Wild animal movement is inherently complex, and the tasks required for survival and reproduction vary widely across species. To better understand these dynamics, future research would benefit from a multivariate analysis of performance traits. Such analyses should adopt a (co)variance partitioning approach that partitions phenotypic (co)variation in performance traits at the among- and within-individual levels and across multiple temporal scales (Careau and Wilson, 2017a,b; Lailvaux et al., 2019).

### Trait repeatability

With high recapture rates, we had a unique opportunity to quantify the repeatability of performance in a mammal across its lifetime and during rapid changes in growth, maturation and senescence. We assessed the repeatability over days, seasons (pre-breeding, breeding, post-breeding), years (2012–2014) and lifetimes. Body size, mass, condition, sprint speed and bite force were moderately to highly repeatable (0.55–0.99) across short-term scales but became more variable over the lifetime of individuals (0.02–0.95). For females, the consistent repeatability of sprint speed across most scales indicates that measurements taken on females of any age in any part of the breeding season were similar to measurements taken on that same individual throughout the entire study (Berberi and Careau, 2019). Therefore, the variance in repeatability over time reflects the natural effects of ageing on morphology and performance, while high repeatability potentially indicates an adaptability to the changes in physiology associated with breeding and changes in environmental conditions (e.g. rainfall, resource availability) over time.

For males, bite force was highly variable over an individual's lifetime, until body size was removed from the model, which significantly increased long-term repeatability (0.02–0.41). This suggests that bite force is greatly influenced by changes in body size associated with the rapid maturation stage of their ‘live fast, die young’ life history. Finally, changes in sprint speed and bite force within testing days may reflect changes in motivation, as animals rarely behave in the lab consistently, or in ways that reflect their true behaviour in nature (Dingemanse and Dochtermann, 2013; Newar and Careau, 2018). Our study highlights the importance of repeated measures over time to better estimate whole-animal performance.

### Conclusions and perspectives

Our findings provide insight into the sex-specific functional strategies of wild northern quolls and the effects of extreme breeding strategies on whole-animal performance. For technically iteroparous females, large body size and strong fighting capacity are key for defence and resource competition. Semelparous males, however, experience selective pressures that favour body size, strong bite forces and robust locomotor strategies that maximise mating opportunities, at the expense of longevity. The absence of a functional trade-off between sprint speed and bite force suggests these traits can evolve independently in this population. Finally, our findings underscore the importance of repeated measures and trait repeatability in revealing detailed changes in morphology and performance over individual lifetimes.

## Supplementary Material

10.1242/jexbio.249969_sup1 Supplementary information
