# Supplementary material for: Sex-specific performance, trade-offs and trait repeatability across the lifetime of the world's largest semelparous mammal (Dasyurus hallucatus)
Source: J Exp Biol. 2025 Apr 7;228(7):jeb249969. doi: 10.1242/jeb.249969 (PMC12045626; doi:10.1242/jeb.249969)
Supplement: Supplementary information [file jexbio-228-249969-s1.pdf]

Supplementary Materials and Methods

Sprint Speed

Camera views overlapped to ensure that each run attempt was captured in full, from a standing start to maximum speed (which occurred approximately 150 cm along the track). A 1 m long tape measure was fixed to the rubber floor across the central area to calibrate movement speed of each quoll. A reference point was also placed in between the shoulder blades of each quoll – a 4 × 4 cm piece of masking tape with a black dot, visible from the cameras above. Quolls were held 1.25 m from the start of the racetrack, and when released were immediately chased towards the opposite end, 3.25 m from the start line. This method ensured that a minimum of 4 strides of linear acceleration were achieved within the 2 m field of view of the camera.

Bite Force

Bite force was quantified using a custom-built force-transducer (as per Cameron et al., 2013) consisting of a secured unit of two metal plates (70 × 10 × 2 mm) separated by a third, larger steel metal pivot plate (3 mm thick). The two smaller plates protruded 12 mm beyond the pivot plate, with a strain gauge (RS Electronics, Sydney, Australia) attached via epoxy resin on the top metal plate. The output from the strain gauge was connected to a custom-made Wheatstone bridge linked to a bridge amplifier (ADInstruments, Sydney Australia). A defined biting point and surface was created on the two protruding plates by covering each in three layers of flesh-like tape (Elastoplast, Beiersdorf, North Ryde, NSW, Australia).

Journal of Experimental Biology • Supplementary information

Results

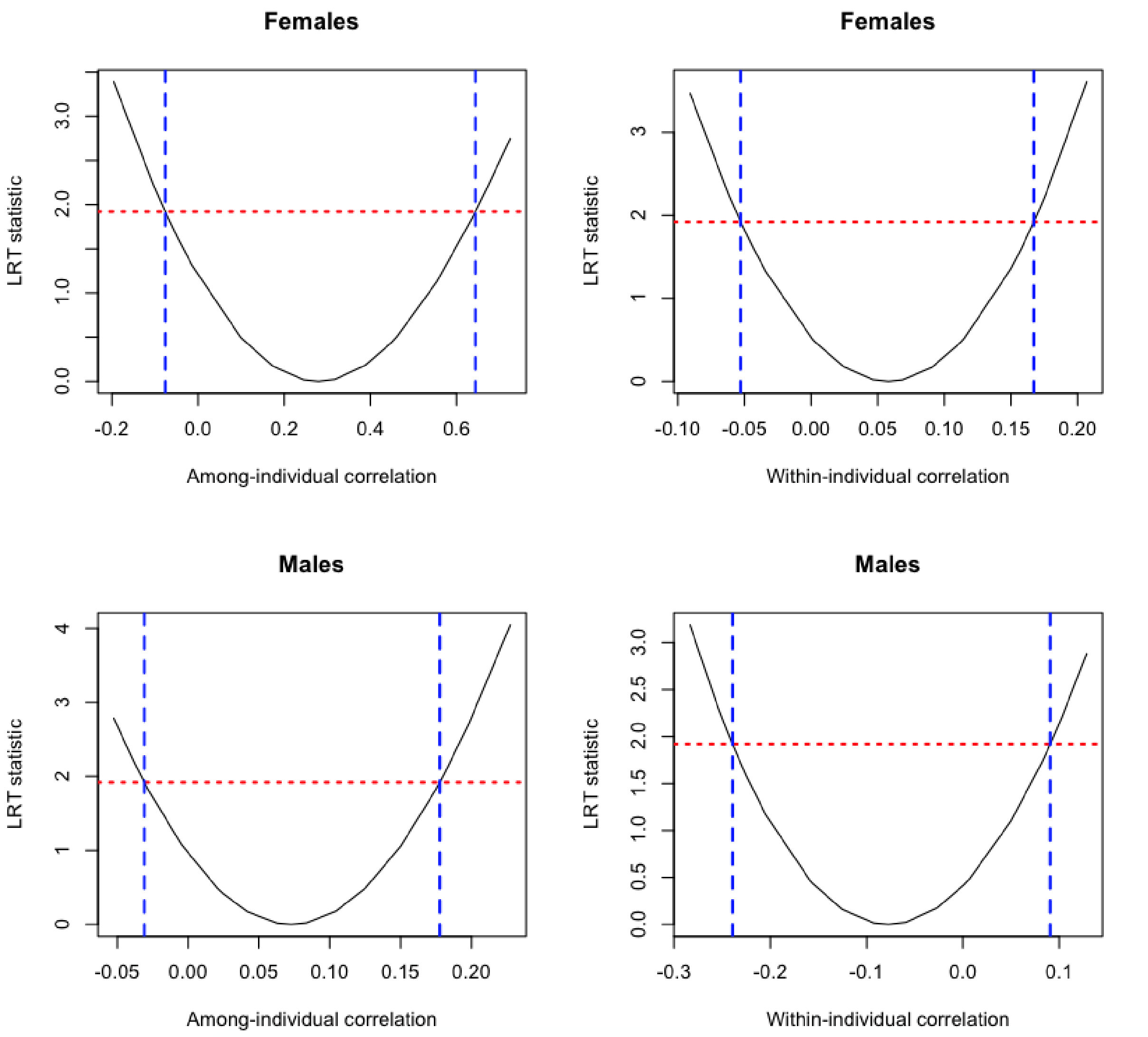

Fig. S1. Plots of profile likelihood tests of significance of among-individual and within-individual correlations between sprint speed and bite force in female and male northern quolls (*D. hallucatus*). Upper and lower 95% confidence intervals are represented by dashed blue lines and the likelihood ratio test (LRT) statistic is marked by the red dashed line. Profile likelihoods were estimated from bivariate mixed models, and calculated using the nadiv package in R. Confidence intervals overlapping 0 indicate non-significant correlations between traits sprint speed and bite force.

Table S1. Table of summary statistics from ANOVA conducted on linear mixed effects model of the effect of reproductive season on sprint speed and bite force in male and female northern quolls (*Dasyurus hallucatus*)

	Sum Sq	Mean Sq	NumDF	DenDF	F value	Pr(>F)
**Sprint Speed**						
Females						
Body Size	0.09	0.09	1	35.34	0.11	0.74
Body Condition	0.44	0.44	1	48.74	0.56	0.46
Season	1.22	0.61	2	40.83	0.77	0.47
Test Number	0.19	0.19	1	47.61	0.24	0.63
Males						
Body Size	0.57	0.57	1	19.29	1.23	0.28
Body Condition	2.15	2.15	1	32.27	4.60	**0.04 ***
Season	4.67	2.33	2	45.59	5.00	**0.01 ***
Test Number	<0.001	<0.001	1	55.22	<0.001	1.00
**Bite Force**						
Females						
Body Size	1.05	1.05	1	35.37	6.82	**0.01 ***
Body Condition	1.23	1.23	1	42.18	8.00	**0.01 ***
Season	1.38	0.69	2	37.21	4.49	**0.02 ***
Test Number	0.07	0.07	1	42.62	0.43	0.52
Males						
Body Size	30.15	30.15	1	23.20	50.02	**<0.001 *****
Body Condition	0.13	0.13	1	72.44	0.22	0.64
Season	1.23	0.62	2	70.84	1.02	0.37
Test Number	0.44	0.44	1	47.87	0.72	0.40

Journal of Experimental Biology • Supplementary information

Table S2. Summary statistics (estimates and 95% CI) from a bivariate mixed model for female northern quolls considering all trials, but retaining only the maximum values for an individual on a given day, of (a) sprint speed and (b) bite force (both standardised to a mean of 0 and a variance of 1).

	**Sprint Speed**				**Bite Force**			
	Estimate		95% CI		Estimate		95% CI	
			Lower	Upper			Lower	Upper
**Females**								
Fixed Effects								
$PC_{bodysize}$	0.01		-0.12	0.13	**0.24**	***	**0.16**	**0.32**
$PC_{condition}$	0.11		-0.08	0.31	**0.22**	*	**0.09**	**0.35**
Age	0.02		-0.36	0.39	0.01		-0.22	0.25
Season		**				**		
Breeding	**-0.36**		**-0.62**	**-0.10**	**-0.43**		**-0.61**	**-0.24**
Post-Breeding	0.04		-0.36	0.44	0.12		-0.15	0.39
Test Number	-0.07		-0.15	0.01	0.02		-0.03	0.07
Variance Components								
V_{ind}	0.30		0.12	0.47	0.04		-0.02	0.10
V_{e}	0.66		0.51	0.81	0.37		0.29	0.45
Repeatability (R)	0.31		0.15	0.46	0.11		-0.04	0.25
Correlation [95% CI]								
Among-individual [r_{ind}]				0.18 [–0.20;0.57]				
Within-individual [r_{e}]				0.07 [–0.05;0.18]				
**Males**								
Fixed Effects								
$PC_{bodysize}$	0.07		0.01	0.13	**0.23**	***	**0.18**	**0.27**
$PC_{condition}$	0.10		-0.08	0.27	0.06		-0.09	0.20
Age						*		
Season	-0.05		-0.34	0.23	-0.16		-0.45	0.12
Breeding	-0.07		-0.62	0.48	0.37		-0.15	0.88
Post-Breeding	**-0.19**	*	**-0.35**	**-0.03**	-0.06		-0.20	0.08
Test Number	0.10		-0.08	0.27	0.06		-0.09	0.20
Variance Components								
V_{ind}	0.56		0.33	0.79	0.03		-0.13	0.18
V_{e}	0.41		0.28	0.53	0.68		0.48	0.87
Repeatability (R)	0.58		0.43	0.73	0.04		-0.18	0.25
Correlation [95% CI]								
Among-individual [r_{ind}]				0.25 [-0.07;0.15]				
Within-individual [r_{e}]				-0.04 [–0.20;0.13]				

Fixed effects included are the same as Table 2 and 3, except for run trial number which was not fitted in this model because only a maximum value per session for each trait was taken. Significant fixed effect estimates are bolded; Signif. codes: 0 '***' 0.001 '**' 0.01 '*'. Variance components of the random effects include individual identity (V_{ind}) and residual (V_{e}), as well as among individual (r_{ind}) and within-individual (r_{e}) correlation estimates. Variance components were used to calculate repeatability.

Journal of Experimental Biology • Supplementary information

Table S3. Repeatability estimates (*R*) and 95% confidence intervals (CI) in (a) body size, (b) body mass, and (c) body condition at each temporal scale extracted from univariate models for wild female and male northern quolls (*Dasyurus hallucatus*)

	Body Size			Body Mass			Body Condition		
	Estimate	95% CI		Estimate	95% CI		Estimate	95% CI	
		Lower	Upper		Lower	Upper		Lower	Upper
**Females**									
Variance Components									
V_{ind}	0.66	0.33	0.98	0.10	0.05	0.15	0.16	0.07	0.26
V_{year}	0.33	0.10	0.56	0.01	-0.02	0.04	0.04	-0.04	0.13
V_{season}	0.09	-0.01	0.19	0.05	0.03	0.08	0.08	-0.01	0.18
V_e	0.10	0.02	0.18	0.03	0.01	0.05	0.11	0.03	0.19
Repeatability									
$R_{long\text{-}term}$	0.56	0.35	0.76	0.53	0.35	0.70	0.41	0.21	0.61
R_{year}	0.84	0.78	0.90	0.58	0.44	0.71	0.52	0.37	0.66
R_{season}	0.91	0.84	0.99	0.86	0.76	0.97	0.72	0.51	0.93
$R_{short\text{-}term}$	-	-	-	-	-	-	-	-	-
**Males**									
Variance Components									
V_{ind}	6.46	4.78	8.15	1.27	0.91	1.62	0.35	0.21	0.49
V_{season}	0.26	0.13	0.39	0.01	-0.12	0.14	<0.01	<0.01	<0.01
V_e	0.09	0.02	0.16	0.20	0.06	0.33	0.29	0.20	0.37
Repeatability									
$R_{long\text{-}term}$	0.95	0.93	0.97	0.86	0.81	0.92	0.55	0.41	0.70
R_{season}	0.99	0.98	0.99	0.87	0.77	0.96	0.55	0.41	0.70
$R_{short\text{-}term}$	-	-	-	-	-	-	-	-	-

Repeatability of performance traits at each temporal scale were calculated using variance components extracted from each univariate model. Variance components of the random effects included in the models: individual identity (V_{ind}), age (V_{year} – females only), season (V_{season}), day of test number (V_{day}), and residual (V_e).

Journal of Experimental Biology • Supplementary information

Table S4. Results of linear models testing for significance between females and males in Mass, Body Size (PC1), Body Condition (PC2), Maximum Bite Force (N), and Maximum Sprint Speed (m s^{-1})

	Group Average				
	Females	Males	t value	df	Pr (>|t|)
Mass (g)	337	486	-19.27	516.05	< 0.001
Body Size (PC1)	-1.25	2.03	-24.39	526.34	< 0.001
Body Condition (PC2)	0.12	-0.19	5.99	740.85	< 0.001
Max Bite (N)	77.6	107.8	3.68	287.47	< 0.001
Max Sprint (m s^{-1})	4.17	4.35	1.08	279.73	0.28

Table S5. Summary statistics (estimates and 95% CI) from a univariate mixed model of the effects of fixed variables on body condition ($PC_{condition}$) for male and female northern quolls (*D. hallucatus*).

	Body Condition			
	Estimate		95% CI	
			Lower	Upper
**Fixed Effects**				
$PC_{bodysize}$	**0.17**	***	**0.14**	**0.20**
Sex		***		
Sex_male	-0.11		-0.38	0.15
Season	-0.01	***	-0.05	0.06
Test Sequence	0.01		-0.01	0.04
Sex × Season				
Sex_male × Season	**-0.36**	***	**-0.45**	**-0.26**

Journal of Experimental Biology • Supplementary information
